# Comparison of the effectiveness of three manual physical therapy techniques in a subgroup of patients with low back pain who satisfy a clinical prediction rule: Study protocol of a randomized clinical trial [NCT00257998]

**DOI:** 10.1186/1471-2474-7-11

**Published:** 2006-02-10

**Authors:** Joshua A Cleland, Julie M Fritz, John D Childs, Kornelia Kulig

**Affiliations:** 1 Assistant Professor, Department of Physical Therapy, Franklin Pierce College 5 Chenell Drive, Concord, NH 03301; and Research Coordinator, Rehabilitation Services, Concord Hospital, Concord, NH, USA; 2 Assistant Professor, Division of Physical Therapy, University of Utah, Salt Lake City, UT; Clinical Outcomes Research Scientist, Intermountain Health Care, Salt Lake City, UT, USA; 3 Assistant Professor and Director of Research, US Army-Baylor University Doctoral Program in Physical Therapy, San Antonio, TX, USA; 4 Associate Professor of Clinical Physical Therapy, Department of Biokinesiology and PT, University of Southern California, Los Angeles, CA, USA

## Abstract

**Background:**

Recently a clinical prediction rule (CPR) has been developed and validated that accurately identifies patients with low back pain (LBP) that are likely to benefit from a lumbo-pelvic thrust manipulation. The studies that developed and validated the rule used the identical manipulation procedure. However, recent evidence suggests that different manual therapy techniques may result similar outcomes. The purpose of this study is to investigate the effectiveness of three different manual therapy techniques in a subgroup of patient with low back pain that satisfy the CPR.

**Methods/Design:**

Consecutive patients with LBP referred to physical therapy clinics in one of four geographical locations who satisfy the CPR will be invited to participate in this randomized clinical trial. Subjects who agree to participate will undergo a standard evaluation and complete a number of patient self-report questionnaires including the Oswestry Disability Index (OSW), which will serve as the primary outcome measure. Following the baseline examination patients will be randomly assigned to receive the lumbopelvic manipulation used in the development of the CPR, an alternative lumbar manipulation technique, or non-thrust lumbar mobilization technique for the first 2 visits. Beginning on visit 3, all 3 groups will receive an identical standard exercise program for 3 visits (visits 3,4,5). Outcomes of interest will be captured by a therapist blind to group assignment at 1 week (3^rd ^visit), 4 weeks (6^th ^visit) and at a 6-month follow-up. The primary aim of the study will be tested with analysis of variance (ANOVA) using the change in OSW score from baseline to 4-weeks (OSW_Baseline _– OSW_4-weeks_) as the dependent variable. The independent variable will be treatment with three levels (lumbo-pelvic manipulation, alternative lumbar manipulation, lumbar mobilization).

**Discussion:**

This trial will be the first to investigate the effectiveness of various manual therapy techniques for patients with LBP who satisfy a CPR.

## Background

An intervention commonly used in the treatment of individuals with low back pain (LBP) is thrust manipulation. The *Guide to Physical Therapist Practice *[1] identifies mobilization/manipulation as an intervention appropriate for the care of patients with spinal disorders. Jette and Jette [2] reported mobilization/manipulation was utilized in 35% of over 1000 patients with LBP treated by physical therapists, less than many other interventions with no supporting evidence. Several randomized trials have found manipulation to be more effective than placebo [3-5] or other interventions [6-9]. However other studies have not shown any benefits for manipulation versus other interventions [10-13]. The disparity in the results of clinical trials may be partly attributable to the admission of all patients with LBP without an attempt to *a priori *identify those likely to benefit from the intervention. A few randomized trials have found manipulation to be more beneficial for a sub-group of patients with more acute symptoms [14,15], or more limited straight leg raise range of motion [16]. Each of these studies was published in 1990 or before, and none sought to develop a multi-factorial classification rule that would maximize the prediction of success with manipulation prior to the intervention.

### Identifying the sub-group of patients likely to respond to spinal manipulation

The classification process can be used to identify patients who will respond favorably to a specific type of treatment based on clusters of signs and symptoms identified during the physical examination. Clinical prediction rules (CPR) are useful to improve the accuracy of the classification of patients. Clinical prediction rules are developed by examining multiple factors from the history and physical examination. Through statistical analysis the most powerful set of predictor variables is identified to maximize accuracy in predicting response to treatment [17].

Recently, a CPR was developed that identifies patients with LBP who are likely to respond with rapid, and prolonged reductions in pain and disability following spinal manipulation (see Additional file 1) [18]. A favorable response to manipulation was defined as a 50% or greater reduction in self-reported disability occurring over two treatment sessions. The CPR developed from this study of 71 patients with non-radicular LBP involved 5 factors; the duration of current symptoms (less than 16 days), score on the work subscale of the Fear-Avoidance Beliefs Questionnaire [19] (less than 19), hypomobility of the lumbar spine, internal rotation of at least one hip greater than 35 degrees, and symptoms not extending distal to the knee. When four out of the five factors were present, the positive LR was 24.4 (95% CI, 4.6 to 139.4). To put this result in perspective, if it is assumed that about 50% of all patients with non-radicular LBP will respond to the manipulation technique, the likelihood of response increases to 97% in an individual with at least four factors present.

A follow-up study [20] was carried out to determine the validity of the CPR developed by Flynn et al [18]. This study randomly assigned 131 patients to receive a standardized exercise program with or without the manipulation technique. Outcomes were assessed at 1 week then at the end of treatment (4 weeks) and at a 6-month follow up. Following data collection a therapist who was blind to treatment allocation determined if the patient satisfied the CPR. The results demonstrated that patients who were positive on the rule and received manipulation experienced greater improvement in 1- and 4-week disability than patients who were negative on the rule and received manipulation. This difference was maintained at the 6-month follow-up. Furthermore, patients who were positive on the rule and received manipulation also experienced greater improvement in 1- and 4-week disability than patients who were positive on the rule but received the exercise. At the 6-month follow-up, patients in the exercise group demonstrated statistically significantly greater medication use, health care utilization, and lost work time due to back pain than patients in the manipulation group. In the manipulation group, positive status on the rule resulted in a positive likelihood ratio of 13.2 (CI, 3.4 to 52.1) for predicting success at 1 week.

#### Generalizability of the manipulation clinical prediction rule

The CPR developed to predict favorable response to manipulation investigated only one manipulation technique, and predominantly used therapists working within the United States Armed Forces. The accuracy of the CPR for predicting the outcomes with therapists in other work settings, or using other manipulation techniques is not known. However, recently, Cleland et al [21] utilized a different manipulation technique than that used in the development of the rule in a consecutive series of patients who satisfied the CPR. Eleven of the 12 patients (92%) in this case series that satisfied the CPR and were treated with an alternative lumbar manipulation technique demonstrated a successful outcome (> 50% reduction in the OSW) in 2 visits. It is plausible that patients with LBP who satisfy the CPR may obtain a successful outcome with different manipulation techniques directed at the lumbopelvic region. However, the inherent limitation of a case series does not allow us to infer a cause and effect relationship between the manipulation and the patient's outcomes. Although some clinicians have argued for superior efficacy with certain techniques recent evidence questions this belief. Chiradejnant et al [22] examined 140 patients with LBP and found no difference in outcomes between patients who received the mobilization technique selected by the treating therapist or a randomly selected technique. Another debate among clinicians is the need to perform thrust manipulation techniques versus non-thrust mobilization techniques. A recent review by Bronfort et al [23] examined the evidence for both thrust manipulation and non-thrust mobilization and concluded that the evidence favors the use of manipulation, particularly for patients with acute LBP, but more studies are needed comparing the different forms of treatment.

The purpose of the present study is therefore to conduct a multi-center randomized clinical trial to compare the outcomes of three different manual therapy techniques in patients with LBP who fit the spinal manipulation CPR. The three techniques used will be; 1) the thrust manipulation technique used in the development of the CPR, 2) an alternative lumbar thrust manipulation technique, and 3) a non-thrust lumbar mobilization technique. A variety of clinicians in geographically diverse practice settings will be used to permit comparison of outcomes within different practice settings.

## Methods

The Institutional Review Boards at Concord Hospital, Concord, New Hampshire and at Intermountain Healthcare Urban Central Region, Salt Lake City Utah have granted approval for the study.

### Study sample

**Figure 1:**
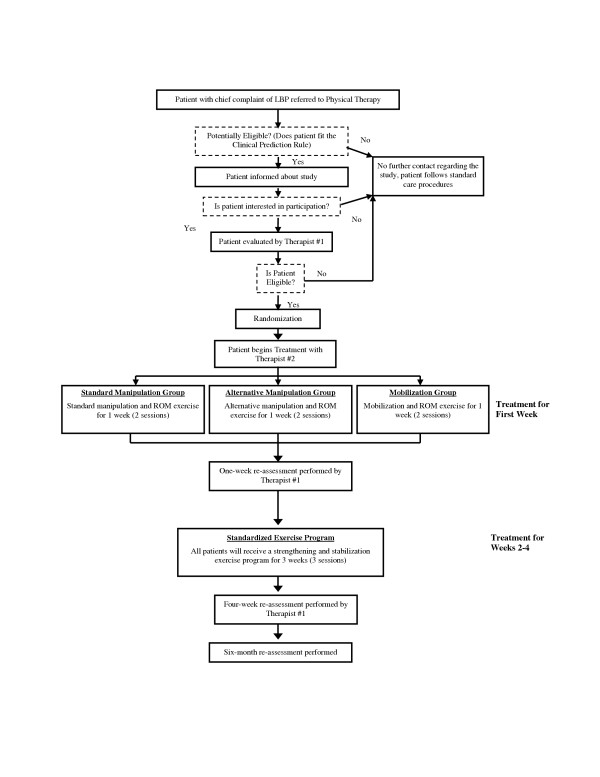
Flow chart demonstrating patient recruitment, study design and timing of data collection.

Two hundred forty subjects with LBP will be recruited from physical therapy clinics in four different geographical locations (60 subjects per location). These sites will provide a diversity of geographic and practice settings. A flow chart demonstrating patient recruitment, study design and timing of data collection can be seen in Figure 1.

The study will include patients who meet the following inclusion criteria:

**1**. Chief complaint of pain and/or numbness in the lumbar spine, buttock, and/or lower extremity

**2**. Oswestry disability score (OSW) of at least 25%

**3**. Age greater than 18 years and less than 60 years

**4**. At least four out of five of the following criteria:

**a**. Duration of current episode < 16 days (judged from the patient's self-report)

**b**. No symptoms extending distal to the knee (judged from the pain diagram)

**c**. Fear-Avoidance Beliefs Questionnaire work subscale (FABQ-W) score <19

**d**. At least one hip with >35° internal rotation range of motion (measured in prone)

**e**. Stiffness in the lumbar spine (judged from segmental mobility testing)

The following exclusion criteria will be used for this study:

**1**. Red flags noted in the participant's general medical screening questionnaire (i.e. tumor, metabolic diseases, RA, osteoporosis, prolonged history of steroid use, etc.)

**2**. Signs consistent with nerve root compression, this includes *any one*of the following:

**a**. Reproduction of low back or leg pain with straight leg raise at less than 45°

**b**. Muscle weakness involving a major muscle group of the lower extremity

**c**. Diminished lower extremity muscle stretch reflex (Quadriceps or Achilles tendon)

**d**. Diminished or absent sensation to pinprick in any lower extremity dermatome

**3**. Prior surgery to the lumbar spine or buttock

**4**. Current pregnancy

**5**. Past medical history of osteoporosis or spinal compression fracture

**6**. Inability to comply with treatment schedule (weekly sessions for four weeks)

#### Examination procedures

All patients meeting the inclusion/exclusion criteria will be eligible for participation. Patients who agree to participate will sign an informed consent document approved by the Institutional Review Board at the respective study site. A therapist blind to group assignment will perform all evaluation procedures. After obtaining informed consent, all subjects will then complete the remainder of the self-report questionnaires and a physical examination. The following self-report questionnaires will be completed by the patient at the baseline examination, demographic data, Numeric Pain Rating Scale (NPRS) [24], Pain Diagram [25], Oswestry Disability Questionnaire (OSW) [26], Fear-Avoidance Beliefs Questionnaire Work (FABQ) [19], Patient Global Rating of Change (GROC) [27]. Following completion of self-report measures the patient will undergo a standardized historical and physical examination. The questionnaires and the physical examination will be repeated following completion of the four-week treatment program. The OSW, NPRS, and GROC will also be completed after the first week (2 sessions) of treatment and at a 6-month follow-up.

#### Randomization

A random number generator will be used to establish randomization lists prior to the initiation of the study. An individual not involved with data collection will generate separate randomization lists for each participating site. Once the baseline examination is completed, a second therapist blind to the baseline examination will open the randomization envelope indicating the patient's treatment group assignment that corresponds to the patient's unique identification number. Patients will receive treatment according to their group assignment for 2 sessions within the first week. Following this all subjects will continue with physical therapy treatment for 3 additional visits (once weekly for 3 weeks) with a standardized exercise regimen.

#### Blinding

Due to the nature of this study, it is not possible to blind the patient or the treating therapist to the treatment received. We will blind the examining therapist performing the baseline and outcome assessments. The examining and treating therapists will be different individuals and the examining therapist will remain blind to the patient's treatment group assignment at all times. Patients will be instructed not to discuss the particular manual therapy technique received with the examining therapist.

#### Treatment

Regardless of treatment group, all patients will be scheduled for the first treatment session within 3 days of the baseline examination. All patients will attend 2 therapy sessions in the first week, then 1 session per week for the next 3 weeks for a total of 5 treatment sessions. In each treatment group, the first 2 sessions will begin with delivery of the randomly assigned manual therapy technique followed by a range of motion exercise. The final 3 sessions will involve instruction in a standardized exercise program. The only difference among the 3 groups will therefore be the type of manual therapy technique used in the first 2 sessions. Otherwise the 3 groups will receive the identical treatment.

#### Manual therapy technique for sessions 1 & 2

##### Standard manipulation group

**Figure 2:**
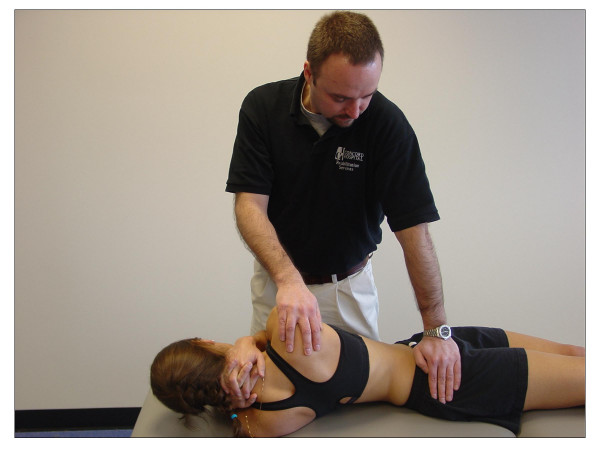
Thrust manipulation technique used in the development and validation of the CPR.

This treatment group will receive the manipulation technique that was used in the development of the CPR [18,20]. The manipulation technique is performed with the patient supine. The therapist stands on the side opposite of that to be manipulated. The patient is passively moved into side-bending towards the side to be manipulated. The patient interlocks the fingers behind his or her head. The therapist passively rotates the patient, and then delivers a quick thrust to the ASIS in a posterior and inferior direction (Figure 2).

We will use the same manipulation decision-making used in validation of the CPR.^34 ^The side to be manipulated will be the more symptomatic side based on the patient's self-report. If the patient cannot specify a more symptomatic side, the therapist may select either side for manipulation. After the manipulation is performed, the therapist will note whether or not a cavitation (ie "a pop") was either heard or felt by the therapist or patient. If a cavitation is experienced, the therapist will proceed to instruct the patient in the ROM exercises. If no cavitation is produced, the patient will be repositioned and the manipulation will be attempted again. If no cavitation is experienced, the therapist will attempt to manipulate the opposite side. A maximum of two attempts per side will be permitted. If no cavitation is produced the therapist will proceed to instruct the patient in the ROM exercises.

##### Alternate side-lying manipulation group

**Figure 3:**
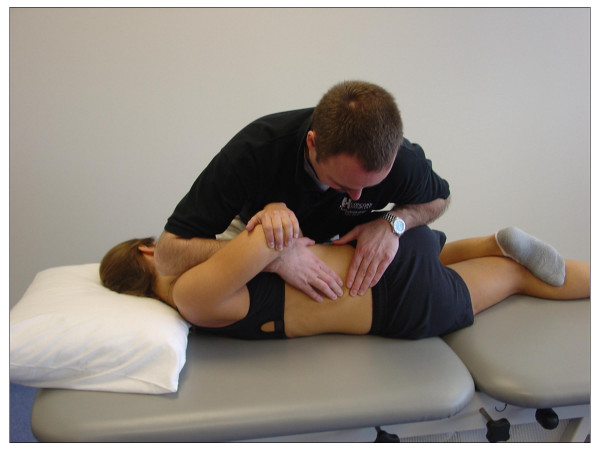
Alternative thrust manipulation technique used in this study.

This treatment group will receive an alternative manipulation technique performed with the patient side-lying. The patient will lie with the more painful side up. The therapist stands in front of the patient. The therapist will then flex the top leg until there is movement at the selected segment (eg. L4–L5) interspace and place the patient's foot in the popliteal fossa of the bottom leg. Next the therapist grasps the patient's bottom shoulder and arm and introduces left trunk sidebending and right rotation until motion is felt at the L4–L5 interspace. The therapist's right thumb is then placed on the right side of the L4 spinous process and the patient's arms are positioned around the therapist's right arm. Setup is maintained while the patient is rolled toward the therapist. Finally the therapist's left arm is used to apply a high velocity, low amplitude thrust of the pelvis in an anterior direction (Figure 3).

We will use the same manipulation decision-making for this group. The side to be manipulated first will be the more symptomatic side based on the patient's self-report. If the patient cannot specify a more symptomatic side, the therapist may select either side for manipulation. The therapist will select the spinal level towards which to direct the manipulation based on segmental mobility assessment performed in side-lying or prone. The therapist will chose a segment in the lower lumbar region towards which to direct the manipulation because the lower lumbar spine is more frequently the source of symptoms in patients with LBP, and recent research suggests greater benefits from manual therapy techniques directed towards the lower lumbar spine [22]. In addition, although this manipulation technique is theoretically directed towards a specific lumbar level, recent evidence suggests that the effects are likely not level-specific [28].

Identical to the standard manipulation group, the therapist will note whether or not a cavitation (ie "a pop") was either heard or felt by the therapist or patient after the manipulation is performed. If a cavitation is experienced, the therapist will proceed to instruct the patient in the ROM exercises. If no cavitation is produced, the patient will be repositioned and the manipulation will be attempted again. If no cavitation is experienced, the therapist will attempt to manipulate the opposite side. A maximum of two attempts per side will be permitted. If no cavitation is produced the therapist will proceed to instruct the patient in the ROM exercises. Following the manipulation treatment, all patients will be instructed in the ROM exercises.

##### Mobilization technique group

This treatment group will receive lumbar postero-anterior mobilizations directed at two spinal segments selected by the treating therapist lumbar segments (eg. L4 and L5). Patients receiving this technique will lie prone. The treating therapist will place the hypothenar eminence of one hand over the spinous process of L4. With the elbows remaining extended, the therapist will deliver oscillatory mobilization (grade IV) for 60 seconds (approximately 30 oscillations) [22,29]. Following a 30 second rest the therapist will again repeat the mobilizations (grade IV) directed at L5 for 60 seconds (approximately 30 oscillations) [22,29]. Following another 30 second rest break rest break the therapist will repeat the procedures at L4 and L5 again.

No cavitation is expected to occur in the mobilization group, however if a cavitation is experienced it will be noted by the therapist. Following completion of the mobilization treatment, the therapist will proceed to instruct the patient in the ROM exercises.

#### Procedures common to all groups

##### Range of motion exercise (visits 1–2)

After receiving the manual therapy intervention, patients in all treatment groups will be instructed in a pelvic tilt range of motion (ROM) exercise. The pelvic tilt exercise will be completed in the physical therapy clinic immediately after the manipulation. Subjects are asked to lie on their back and bend the hips and knees so that their feet are flat on the surface. Subjects then attempt to flatten their back on the table by slightly "drawing in" their stomach and rotating the hips backwards without holding their breath. The motion is to be performed in a pain-free range. Subjects will be instructed to perform a set of 10 repetitions in the clinic during the first and second sessions after manipulation and will be instructed to perform 10 repetitions of the exercise 3–4 times daily until the beginning of the third treatment session. Subjects' will self-report their compliance with range of motion exercise program in an exercise log. At the beginning session #3, the therapist will instruct subjects in this group in the standardized exercise regimen.

##### Standardized exercise program (visits 3–5)

Beginning on the third treatment session, all patients will be treated with the same strengthening and stabilization exercise program. The program is based on current evidence from the biomechanical literature [30-33]. The AHCPR Clinical Practice Guidelines for Adults with LBP [34] recommends muscle strengthening exercises for patients with acute LBP, and evidence also supports exercise therapy for individuals with chronic LBP [35]. The exercise program will target the trunk musculature that has been identified as important stabilizers of the spine including transversus abdominus, oblique abdominals, and the multifidus/erector spinae [30-33]. Patients will also be asked to complete the strengthening program once per day on the days they do not complete the exercise program during the physical therapy session. Patients will self-report their compliance with the exercise program in an exercise log.

### Data analysis

We will recruit 60 patients per clinical site, for a total enrollment of 240 patients across the four geographical locations. A sample size of 240 patients will provide 93% power to detect a minimal difference of 5 points on the OSW assuming a common standard deviation of 10 points. A sample size of 60 within each geographical location will provide 83% power to detect a difference of 9 points on the OSW assuming a standard deviation of 10 points. This sample size will provide sufficient power to detect a minimum clinically important difference in the entire sample, and a moderate difference within a geographical location.

Descriptive statistics, including frequency counts for categorical variables and measures of central tendency and dispersion for continuous variables will be calculated to summarize the data. Baseline demographic data will be compared across treatment groups to assess the adequacy of the randomization. If appropriate, statistical adjustments will be made for baseline characteristics that are significantly different between groups. An intention-to-treat analysis will be utilized in which all participants will be analyzed in the group to which they were originally assigned. All drop-outs and the reason for dropping out of the study will be reported. An *a priori *alpha level of 0.05 will be used for all analyses. All data will be screened to ensure they meet the assumptions for the inferential statistical analyses described below. If they do not meet the necessary assumptions, appropriate non-parametric procedures will be utilized.

The primary aim will be tested with analysis of variance (ANOVA) using the change in OSW score from baseline to 4-weeks (OSW_Baseline _– OSW_4-weeks_) as the dependent variable. The independent variable will be treatment with three levels (lumbo-pelvic manipulation, alternative lumbar manipulation, lumbar mobilization). A second ANOVA will be conducted on the one-week OSW change scores to test for the presence of an immediate treatment effect. We will also repeat the ANOVA procedure for the 6-month follow up outcomes, and using pain rating as the dependent variable.

## Discussion

We have presented the rationale for the need to determine the effectiveness of various thrust and non-thrust manual therapy techniques in patients with LBP who satisfy a CPR. The design we selected will allow for the analysis of the primary outcome measure (OSW) at 1 4 week, 4 weeks and at a 6-month follow-up. Results of this trial will be disseminated as soon as they become available.

## Competing interests

The author(s) declare they have no competing interests.

## Authors' contributions

JAC, JMF and JDC were responsible for the initial conception of the research question. JAC and JMF were responsible for designing the study and developing the protocol. JDC and KK provided a critical review of the proposal prior to completion. All authors are acting as trial coordinators at their respective geographical locations. All authors have read and approved the final manuscript.

## Pre-publication history

The pre-publication history for this paper can be accessed here:



## Supplementary Material

Additional File 1 Video describing the development and validation of the CPR. This video describes the clinical prediction rule for identifying patients with low back pain that are likely to respond to rapidly and dramatically to spinal manipulation. Click here for file
