# Supplementary material for: Comparison of the effectiveness of three manual physical therapy techniques in a subgroup of patients with low back pain who satisfy a clinical prediction rule: Study protocol of a randomized clinical trial [NCT00257998]
Source: BMC Musculoskelet Disord. 2006 Feb 10;7:11. doi: 10.1186/1471-2474-7-11 (PMC1421401; doi:10.1186/1471-2474-7-11)
Supplement: Additional File 1 — Video describing the development and validation of the CPR. This video describes the clinical prediction rule for identifying patients with low back pain that are likely to respond to rapidly and dramatically to spinal manipulation. [file 1471-2474-7-11-S1.zip › embed.html]

 Dr. Tim Flynn -- Clinical Prediction Rule -- Regis University

|  |
| --- |
|  |
